# Disseminated Intravascular Coagulation With Excessive Fibrinolysis Following Diagnostic Prostatic Biopsy for Prostate Cancer

**DOI:** 10.7759/cureus.30502

**Published:** 2022-10-20

**Authors:** Muhammad Ghallab, Usman Ilyas, Lilian Tran, Toka Amin, Salma Abdelmoteleb

**Affiliations:** 1 Internal Medicine, Icahn School of Medicine at Mount Sinai, New York, USA; 2 Internal Medicine, Queens Hospital Center, New York, USA; 3 Internal Medicine, Cairo University School of Medicine, Cairo, EGY

**Keywords:** prostate cancer, primary fibrinolysis, hyperfibrinolysis, secondary fibrinolysis, patent foramen oval, epsilon aminocaproic acid, disseminated intravascular coagulation (dic), excessive fibrinolysis

## Abstract

The most common hematological disorder associated with prostate cancer is disseminated intravascular coagulation (DIC). In most cases, cancer patients with DIC have compensated fibrinolysis with a low incidence of bleeding. However, DIC with excessive fibrinolysis is a rare albeit life-threatening variant that can present with massive bleeding and is thought to occur due to the unique properties of neoplastic cells of prostate cancer that activate both procoagulant and anticoagulant pathways simultaneously. Depending on the shift of the intricate balance between the two forces, the net result can be either systemic micro- (DIC) or macro-thrombi, deep venous thrombosis (DVT) or pulmonary embolism, or a bleeding syndrome from excessive vicious activation of fibrinolysis.

Here, we present a unique case of suspected prostate cancer who underwent a diagnostic prostatic biopsy. Subsequently, he developed massive hematuria requiring intensive care unit admission with multiple supportive blood products. Additionally, he was administered epsilon-aminocaproic acid with a prophylactic dose of heparin, with prompt resolution of bleeding. After stabilization, he was discharged with planned outpatient chemotherapy. However, he subsequently presented with lower extremity DVT within a week, which led to a stroke in the setting of a patent foramen ovale. This unique case report highlights how a change in the intricate balance of the coagulation cascade causes a polar shift in clinical presentation and complications.

## Introduction

Fibrinolysis is the process of breaking down fibrin to maintain the patency of the microvasculature. The process is balanced by a subset of pro- and anticoagulation factors, of which inherited or acquired disturbances can cause a spectrum of potentially life-threatening thrombotic and bleeding disorders [1,2]. Disorder of fibrinolysis is a notorious paraneoplastic phenomenon substantially noted in prostate cancer. This is attributed to the profuse pathologic release of abundantly existing profibrinolytic factors from the distorted architecture of the prostate gland, especially following surgical manipulation [3]. Secondary hyperfibrinolysis due to consumptive disseminated intravascular coagulation (DIC) is the most notable and reported coagulopathy in prostate cancer, whereas primary hyperfibrinolysis is very rare [4]. On the other hand, venous thromboembolism (VTE) is a common paraneoplastic association and cause of death in cancer patients, but its risk is low in prostate cancer [5]. Despite that fact, VTE has recently been reported as a frequent complication following radical prostatectomy [3]. We report a rare case of paraneoplastic hyperfibrinolysis complicated with postoperative bleeding and extensive venous thrombosis in a newly diagnosed prostate cancer patient.

## Case presentation

**Figure 1:**
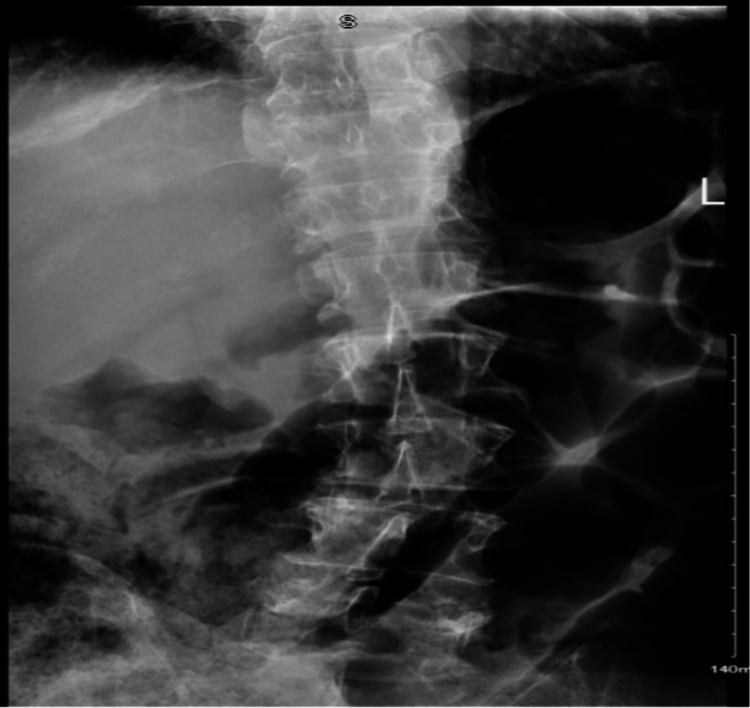
An x-ray of the lumbar spine was ordered and showed levoscoliosis with evidence of osteoarthritis and degenerative disc disease in L3-L4, L4-L5, and L5-1 vertebrae

A 57-year-old Georgian male with no known past medical history presented to our emergency department with low back pain for the past year. He described the pain as intermittent and worse with movement but was stable over the years. The patient tried ibuprofen and acetaminophen with partial improvement. The patient worked as a furniture mover for many years in the past but quit two months ago. He had not seen a primary care physician for many years and never had a prostate-specific antigen (PSA) test or digital rectal exam. The patient's father died of prostate cancer and was diagnosed at age 65. Otherwise, he denied any other symptoms of fever, chills, cough, dysuria, urinary frequency or urgency, headaches, dizziness, or syncope. Upon exam of the lumbar spine, there was decreased range of motion due to pain and paraspinal tenderness. Otherwise, it was unremarkable. An x-ray of the lumbar spine (Figure 1) was ordered and showed levoscoliosis with evidence of osteoarthritis and degenerative disk disease in L3-L4, L4-L5, and L5-S1, with no evidence of fracture. The pain improved with intravenous ketorolac and methocarbamol, and the patient was discharged with a follow-up appointment in our primary care clinic.

Two weeks later, labs were ordered and reviewed at the clinic, including complete blood count (CBC), comprehensive metabolic panel, hemoglobin A1c, lipid panel, syphilis screening, and PSA. The patient was found to have a total PSA level of 542 ng/mL (reference range: 0.0-4.0 ng/mL), free PSA > 50 ng/mL, and PSA free % could not be calculated. His hemoglobin level was 11.8 g/dL (14.0-18.0 g/dL). A urology appointment was made for the following week.

**Figure 2:**
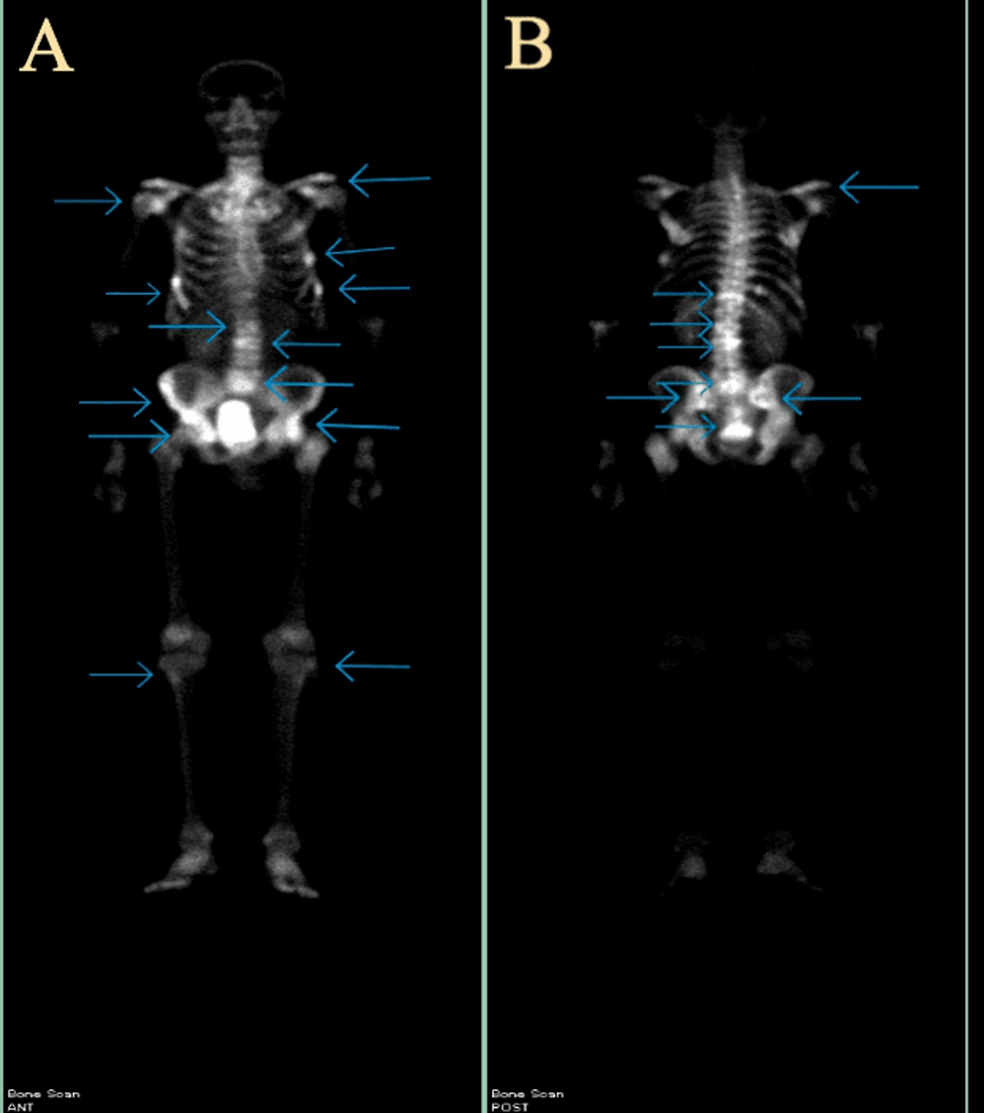
Nuclear medicine whole-body bone scan demonstrates abnormal uptake (arrows) in the right humeral head, several ribs bilaterally, sacroiliac joint bilaterally, tibial tuberosities bilaterally, pelvic and pubic bones bilaterally, linear uptake in T12, and abnormal uptake in L3-L4 and L5-S1 intervertebral joints. The findings are consistent with metastatic skeletal disease, compression fracture in T12, and severe arthritic changes in the lumbar vertebral bodies. Images A and B show anterior and posterior views, respectively. Bright areas show abnormal uptake in the respective bones of the skeleton, consistent with metastatic disease. ANT: Anterior view; POST: Posterior view.

During his urology appointment, the patient admitted to having constipation that he treated intermittently with stool softener as well as nocturia and incomplete emptying but denied gross hematuria. A CT abdomen and pelvis with contrast and nuclear medicine whole-body bone scan (Figure 2) were ordered for further evaluation.

**Figure 3:**
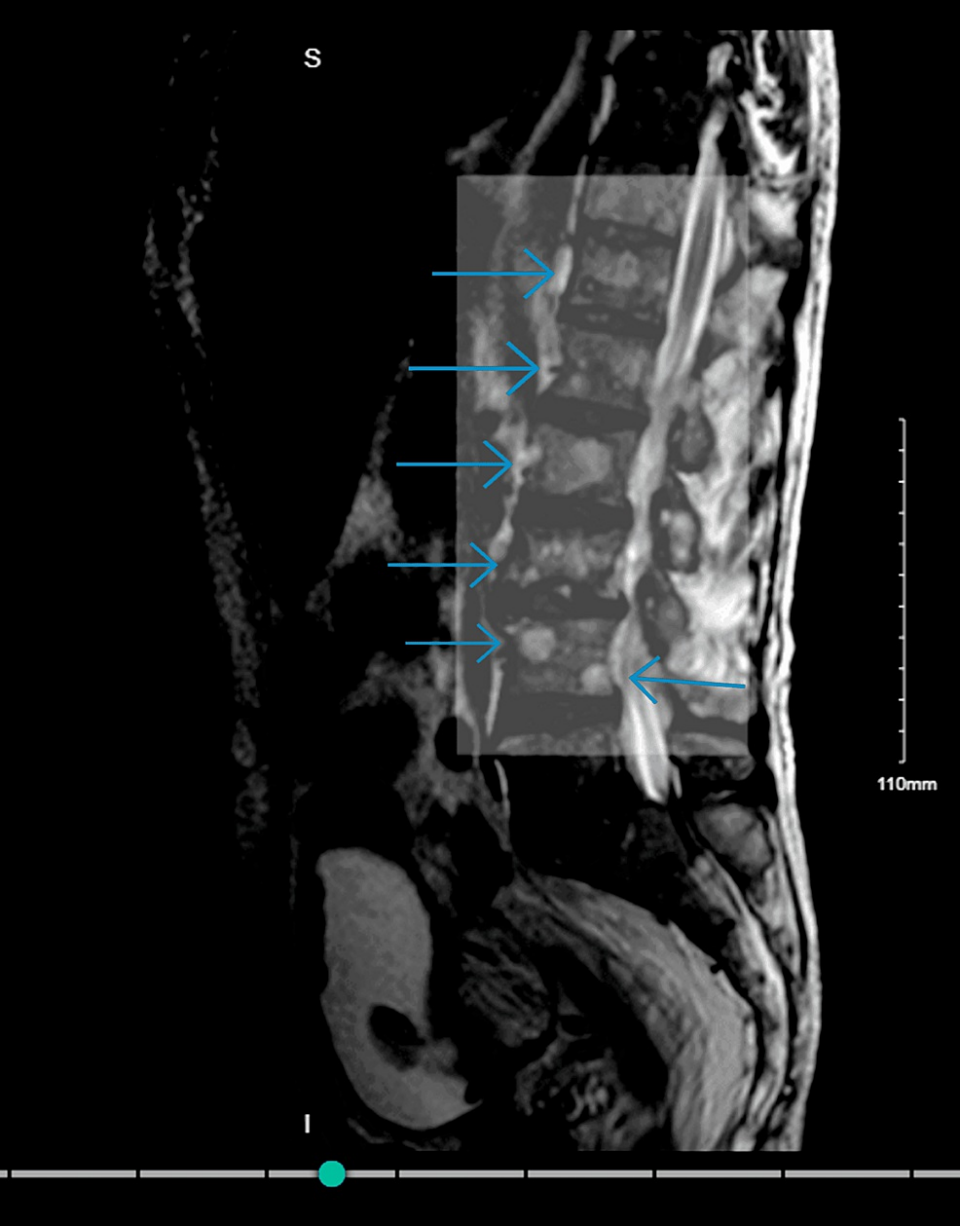
Magnetic resonance imaging of the spine shows multiple metastatic lesions (highlighted area)

An appointment was also made for a transrectal ultrasound-guided prostate biopsy procedure. The patient was diagnosed with benign prostatic hyperplasia and was started on finasteride, tamsulosin, and docusate for constipation. Approximately two weeks later, during the postoperative period from the biopsy, the patient attempted to urinate but voided only 20 mL of bloody urine. A bladder scan was done, which noted 450 mL of urine. A foley catheter was inserted and drained about 600 mL of bloody urine. The patient was hypotensive with postoperative CBC showing a drastic drop in platelets to 25 x 10^3^/mcL (150-450 x 10^3^/mcL) and hemoglobin of 8.2 g/dL. The patient was transfused with two units of platelets and two units of packed red blood cells (pRBCs). MRI of the bone and the spine with contrast (Figure 3) was performed, which showed metastatic disease to the left femur, pelvis, and spine.

The following morning, the patient's CBC showed a mild increase in platelets to 36 x 10^3^/mcL with hemoglobin of 7.9 g/dL, fibrinogen levels at 71 mg/dL (200-393 mg/dL), an international normalized ratio of 1.8, and D-dimer > 1000 ng/mL (<285 ng/mL). A hematology consult was placed. The patient denied any history of nosebleeds in the past or bleeding after tonsillectomy or tooth manipulation. Due to the catheter continuously draining bright red blood and lab values, the patient was transfused one unit of cryoprecipitate, one more unit of platelets, and two more units of pRBCs. The patient was also started on aminocaproic acid 1000 mg with an increase in frequency every two hours. He was transferred to the medical intensive care unit with the impression of hematuria and anemia due to blood loss requiring transfusion of multiple blood products secondary to hyperfibrinolysis activated after prostate biopsies.

Other labs were also obtained, including euglobulin clot lysis time > 60 minutes, thromboelastography, urea clot solubility test, von Willebrand factor, factor XIII antigen 13% (51%-163%), factor XIII activity 10% (57%-192%), mixing studies, alpha 2-antiplasmin, plasminogen activator inhibitor 1, thrombin activatable fibrinolysis inhibitor, and tissue plasminogen activator. The patient's peripheral blood smear was also negative for schistocytes. Despite multiple transfusions of pRBCs, platelets, and cryoprecipitate, the patient's hemoglobin was 6 g/dL, platelets were 40 x 10^3^/mcL, and fibrinogen was 126 mg/dL. Over the next four days, he continued to receive aminocaproic acid with improvement in hematuria and stabilization of hemoglobin and platelets to 9 g/dL and 104 x 10^3^/mcL, respectively. However, fibrinogen levels were still unstable (ranging between 114 and 147 mg/dL), requiring cryoprecipitate transfusions. The following day, the patient's hematuria resolved. Urology planned to start leuprorelin in the next two days. CBC and fibrinogen levels were monitored every 12 hours to keep fibrinogen greater than 150 mg/dL. The patient was deemed hemodynamically and medically stable to transfer to the medicine floor.

The fibrinogen level was repeated on the floor and found to be 68 mg/dL. He was transfused one unit of cryoprecipitate. Afterward, fibrinogen levels remained stable. The patient's foley catheter was removed with no urine retention. As his hemoglobin, platelet, and electrolyte levels were stabilized, he was safely discharged with a follow-up with hematology/oncology and urology.

**Figure 4:**
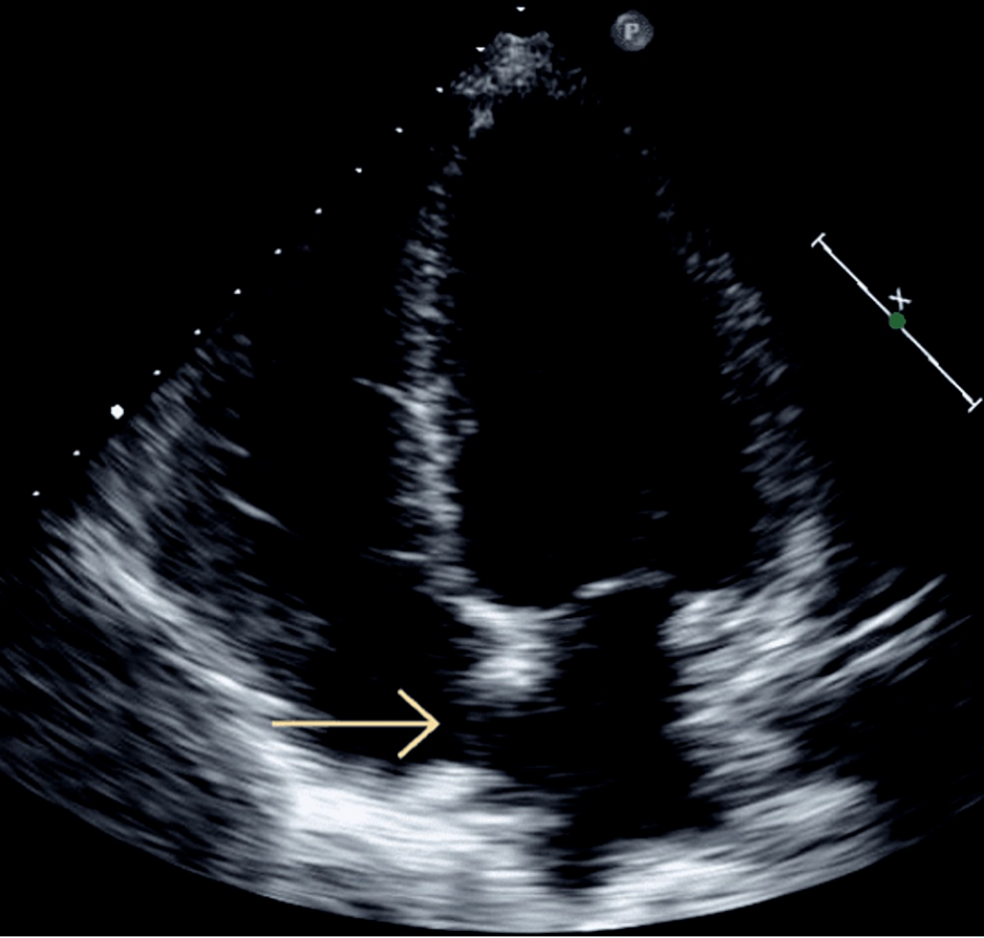
Transthoracic echocardiography showing large patent foramen ovale (pale yellow arrow)

**Figure 5:**
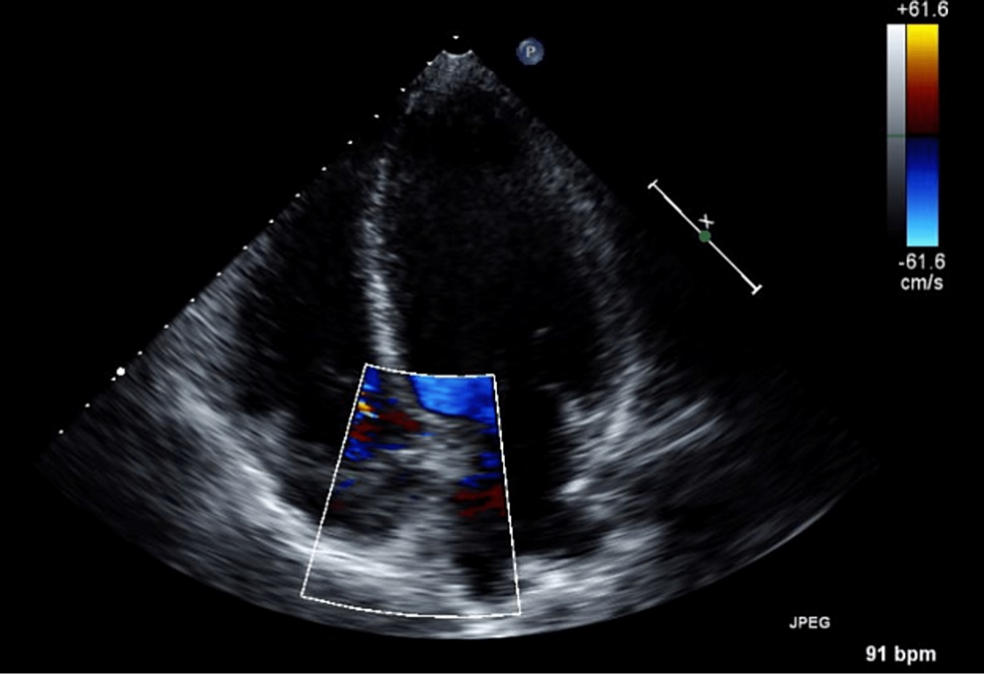
Transthoracic color Doppler echocardiography showing flow across suspected patent foramen ovale

Six days later, the patient returned to the hospital and was admitted to evaluate for left-sided leg pain and swelling for the past week. The patient described the pain as dull, worsening to 10/10, with swelling and disturbed sleep. Otherwise, he denied any shortness of breath or chest pain. Lower extremity duplex ultrasound showed noncompressible echogenic thrombus within dilated common femoral/great saphenous, proximal superficial femoral, mid superficial femoral, distal superficial femoral, and popliteal veins. A hematology service was consulted and recommended therapeutic enoxaparin. The patient was discharged within five days with a follow-up in the pain management clinic the following day. Four days later, the patient returned to the emergency department for slurred speech and facial droop that started around 8 am. He denied any other complaints at that time. The patient was evaluated by neurology when the decision was made to initiate dual antiplatelet therapy for stroke/transient ischemic attack. The patient underwent an MRI of the brain without contrast, which showed acute to subacute stroke of the left and right posterior cerebellum. The patient also had elevated troponin-T levels of 0.476 ng/mL (<0.010 ng/mL), and the electrocardiogram showed sinus rhythm at 71 beats per minute with no ischemic changes. The patient was deemed not a candidate for tissue plasminogen activator (TPA) treatment, given that he was taking therapeutic enoxaparin for deep venous thrombosis (DVT). He was started on dual antiplatelet therapy with Aspirin and clopidogrel. The patient's troponin was later down-trended. He was continued on enoxaparin and was also started on atorvastatin. A transthoracic echocardiogram (Figures 4, 5) showed a moderately dilated left atrium, a moderately dilated right atrium, and severely elevated central venous pressure (11-20 mmHg). A bubble study was significant for sizable patent foramen ovale (PFO).

**Figure 6:**
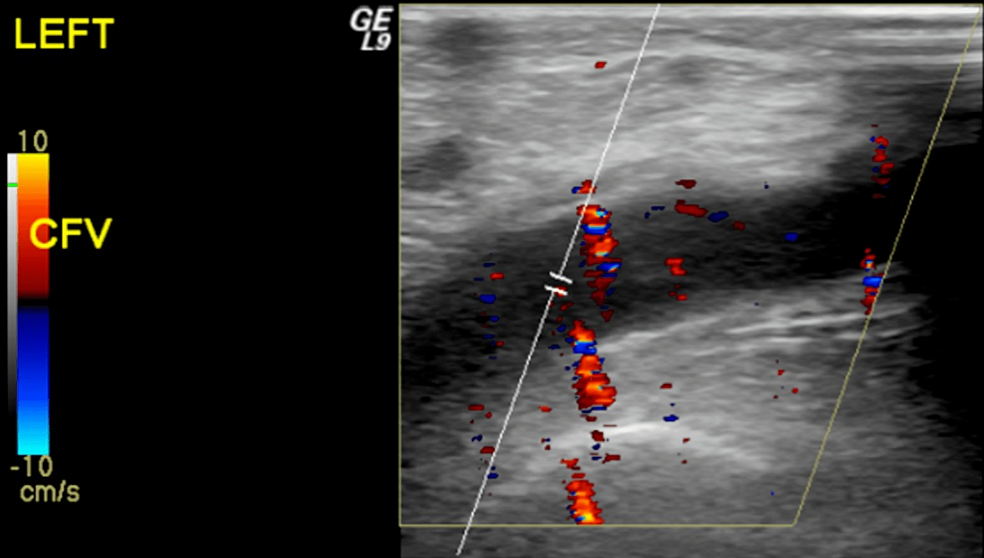
Left lower limb venous duplex showing noncompressible echogenic thrombus within the dilated common femoral vein CFV: Common femoral vein.

Cardiothoracic service was consulted and recommended that there was no indication for closure of PFO, given a low risk of paradoxical embolism (ROPE) score of six and that the patient is already on anticoagulation. The patient was eventually given bicalutamide for malignancy and then started on leuprorelin and enzalutamide. Since then, the patient has had multiple visits to the emergency department due to increasing left lower extremity pain and swelling. A repeat lower extremity duplex (Figure 6) showed acute deep vein thrombosis of the common, deep, and superficial femoral, popliteal, posterior tibial, and peroneal veins.

The patient was evaluated with a CT abdomen pelvis venogram for possible inferior vena cava (IVC) filter placement, which showed left iliofemoral DVT, no definite thrombus within the right iliofemoral veins or IVC, and no other change from prior CT. The patient was discharged three days after his pain was controlled. The decision was made by vascular surgery to rather place an IVC filter; the patient will be placed on rivaroxaban indefinitely.

## Discussion

Hematological disorders associated with prostate cancer include thrombotic thrombocytopenia, thrombosis (DVT and/or pulmonary embolism), primary hyperfibrinolysis, sterile thrombotic endocarditis, acquired factor VIII inhibitor development, and DIC [6]. DIC is the most common disorder observed in prostatic cancer patients [6]. Typically, prostate cancer patients with DIC have compensated fibrinolysis, and the incidence of bleeding is low, around 0.4%-1.65% [7]. However, DIC with excessive fibrinolysis (XFL) is a rare and life-threatening variant of DIC in patients with prostate cancer, manifesting as systemic, intracavitary, intracutaneous, and intracranial bleeding [7].

The mechanisms underlying the association between cancer and DIC are multifactorial and partially understood [8]. The proposed mechanism is the expression of procoagulant molecules, including tissue factor, which activates the hemostatic system, subsequently leading to thrombosis and consumption of the coagulation factors [8]. This theory is further supported by a recent study identifying the release of microparticles, membrane blebs released from the tumor cells' surface, as the coagulation cascade's primary stimulant [9]. These particles provide a surface for assembling coagulation cascade proteases [9]. Activation of hyperfibrinolysis in malignancy occurs in two ways. First, tumor cells produce fibrinolytic system proteins, including the urokinase-type plasminogen activator (uPA) and the tissue-type plasminogen activator [10]. Second, tumor cells carry the specific urokinase plasminogen activator receptor on their membranes, favoring the assembly of the fibrinolytic components, thereby facilitating excessive activation of the fibrinolytic cascade [10].

The uPA is also involved in mediating invasion, metastasis, and modulating cell adhesion, thereby promoting the formation and progression of cancer [11]. Plasmin activity is regulated by the enzymes like a2-antiplasmin, which inactivate plasmin, limiting its action [12]. There is a depletion of this inhibitor in DIC with XFL to levels below 60% of normal, resulting in a bleeding diathesis [12]. uPA catalyzes the conversion of plasminogen into plasmin without negative feedback, resulting in the breakdown of fibrin clots and the degradation of the extracellular matrix [13]. DIC has been reported to occur following a prostate gland biopsy [13]. Clinically, significant bleeding developed in our patient following prostatic biopsy. Since subclinical DIC can be seen in patients with prostatic cancer, less invasive diagnostic approaches or ruling out subclinical DIC before prostatic biopsy should be considered to avoid catastrophic consequences [13]. Additionally, baseline labs should be done before a scheduled procedure to ensure subclinical DIC is not present. In our patient, the last labs were done a month before the biopsy and may have developed subclinical DIC before the biopsy initiated the fibrinolytic cascade culminating in massive hematuria.

In the most extensive retrospective study of prostate cancer with DIC and XFL, features of patients on presentation included metastatic disease (95%), high-grade tumors (>50% with Gleason scores > 7), castration-resistance disease (93%), having received at least two lines of therapy (90%), and previous treatment with taxanes (50%) [7]. Our patient had a Gleason score of 8 and metastatic disease at presentation. Additional points highlighted in that report included bleeding complications seen in around 79% of the patients, intracutaneous (33%), genitourinary (26%), gastrointestinal (18%), and intracranial (28%). The study indicated a median survival of four weeks. In only 18% of the patients, reversal of DIC with XFL occurred, all of whom received chemotherapy and supportive therapy with transfusions. This cohort of patients had an improved median survival of 26 weeks compared to patients who did not achieve a DIC and XFL reversal, highlighting the importance of both prompt recognition and treatment [7].

Primary hyperfibrinolysis implies evidence of systemic activation of plasmin or direct degradation of fibrinogen in the absence of DIC [14]. The clinical and laboratory findings depict the effects of fibrinolysis and fibrinogenolysis [14]. Primary fibrinogenolysis and DIC may be distinguished with a normal platelet count, normal factors V and VIII, markedly low fibrinogen level, negative fibrin soluble monomer complex, and normal level of D-dimer seen in former patients (Table 1) [14]. In DIC, the following results are obtained with decreasing frequency: platelets decreased, fibrin degradation products increased, prothrombin time prolonged, activated partial thromboplastin time prolonged, and fibrinogen decreased [10]. D-dimer is typically highly positive [10]. In addition, on peripheral smears, schistocytes will be seen in DIC and are typically absent in isolated hyperfibrinolysis [15].

**Table 1:** Laboratory parameter differences between disseminated intravascular coagulation and disseminated intravascular coagulation with excessive fibrinolysis AAP: Alpha 2-antiplasmin; DIC: Disseminated intravascular coagulation; FDP: Fibrin degradation product; INR: Internationalized normalized ratio; PAP: Plasmin-antiplasmin complex; PLTs: Platelets; PT: Prothrombin time; PTT: Partial thromboplastin time; TAT: Thrombin-antithrombin complex; uPA: Urinary-type plasminogen activators; XFL: Excessive fibrinolysis.

	DIC	XFL
Elevated	PTT	FDP
			PTT
	INR		PAP
	D-dimer		
	TAT		
	Decreased	Platelets	Fibrinogen
AAP		
Fibrinogen (normal to decreased)		D-dimers/FDP ratio
		Euglobulin lysis time
Positive	Fibrinogen monomer test	Fibrin monomer
		uPA test

The use of epsilon-aminocaproic acid has been associated with catastrophic thrombosis in patients with DIC. Based on the available data regarding prior case reports of isolated hyperfibrinolysis, several authors have concluded that these cases represented DIC with XFL as evidenced by accelerated thrombin generation in most cases. The practical importance is that when epsilon-aminocaproic acid is considered, even with bleeding as a significant clinical problem, a low dose of heparin should be added [16]. Intravenous heparin has been postulated to attenuate bleeding in DIC by reducing clotting factor consumption and mitigating the secondary fibrinolytic process, in addition to inhibiting the activation of coagulation and reducing thrombotic complications [15].

Many authors consider using antifibrinolytic agents, such as tranexamic acid and epsilon-aminocaproic acid, to treat bleeding in the context of DIC, a relative contraindication, as they inhibit the secondary fibrinolytic process and promote clot formation [15]. Antifibrinolytic agents are lysine analogs that prevent plasmin from binding to fibrinogen or fibrin, preventing proteolysis [15]. Tranexamic acid is given orally and has a longer half-life and higher potency effect than epsilon-aminocaproic acid [15]. Recent evidence has come to light regarding successful treatment with antifibrinolytic agents of bleeding complications associated with XFL in acute promyelocytic leukemia and metastatic prostatic cancer [15]. Our patient presented one week after discharge with extensive acute on chronic DVT and later embolic stroke in the setting of PFO, which was later closed, despite receiving a prophylactic heparin dose with epsilon-aminocaproic acid.

Other effective therapies reported in the literature are those that achieve rapid androgen ablation, which is most rapidly achieved via orchiectomy; however, immediate surgery is typically not feasible and is contraindicated in the setting of active bleeding secondary to DIC with XFL [16]. Medical therapies for decreasing circulating androgens include high-dose diethylstilbestrol and ketoconazole and have been described in rapidly correcting coagulopathy in literature [16]. Gonadotropin-releasing hormone (GnRH) agonists have been avoided in the treatment of prostate cancer with XFL due to concerns of an androgen surge, causing deteriorating coagulopathy and additional complications such as cord compression [17]. Chemotherapy is the standard of care for patients who are not responding to hormonal therapies or have a castrate-resistant disease [17].

## Conclusions

Prostatic cancer can be associated with various hematological disorders, which pose a significant challenge in both diagnoses and treatment. A high degree of suspicion and anticipation is required to diagnose it early and treat it aggressively to minimize complications and decompensation. Our case highlights several important considerations. Basic labs before the prostatic biopsy were obtained more than two weeks ago in our patient. He may have developed low-grade DIC in the interim, which decompensated to XFL on manipulation of the prostatic tissue. Although epsilon-aminocaproic acid was used in acute management and given a prophylactic dose of heparin, he subsequently presented the following week with acute DVT, complicated by an acute stroke in the presence of PFO. It is unclear whether this was due to the setting of hypercoagulability from the underlying neoplasm or a complication from the medication. Alternative modes of management, as reported above, should be explored as preferred agents, unless life-saving intervention is needed as epsilon-aminocaproic acid was highly effective in promptly stopping bleed in our patient.

## References

[1] Franchini M, Mannucci PM (2018). Primary hyperfibrinolysis: facts and fancies. Thromb Res.

[2] Kolev K, Longstaff C (2016). Bleeding related to disturbed fibrinolysis. Br J Haematol.

[3] Lippi G, Plebani M, Franchini M, Guidi GC, Favaloro EJ (2009). Prostate-specific antigen, prostate cancer, and disorders of hemostasis. Semin Thromb Hemost.

[4] Smith JA, Jr. Jr., Soloway MS, Young MJ (1999). Complications of advanced prostate cancer. Urology.

[5] Sheth RA, Niekamp A, Quencer KB, Shamoun F, Knuttinen MG, Naidu S, Oklu R (2017). Thrombosis in cancer patients: etiology, incidence, and management. Cardiovasc Diagn Ther.

[6] Cooper DL, Sandler AB, Wilson LD, Duffy TP (1992). Disseminated intravascular coagulation and excessive fibrinolysis in a patient with metastatic prostate cancer. Response to epsilon-aminocaproic acid. Cancer.

[7] Hyman DM, Soff GA, Kampel LJ (2011). Disseminated intravascular coagulation with excessive fibrinolysis in prostate cancer: a case series and review of the literature. Oncology.

[8] Jafri MAS, Cohen JV, Much MA, Petrylak DP, Podoltsev NA (2016). A patient with pancytopenia, intractable epistaxis, and metastatic prostate cancer: how correct diagnosis of primary hyperfibrinolysis helps to stop the bleeding. Clinical Genitourinary Cancer.

[9] Kulić A, Cvetković Z, Libek V (2016). Primary hyperfibrinolysis as the presenting sign of prostate cancer: a case report. Vojnosanit Pregl.

[10] Okajima K, Kohno I, Soe G, Okabe H, Takatsuki K, Binder BR (1994). Direct evidence for systemic fibrinogenolysis in patients with acquired alpha 2-plasmin inhibitor deficiency. Am J Hematol.

[11] Ong SY, Taverna J, Jokerst C (2015). Prostate cancer-associated disseminated intravascular coagulation with excessive fibrinolysis treated with degarelix. Case Reports in Oncological Medicine.

[12] Key NS, Kwaan HC (2010). Microparticles in thrombosis and hemostasis. Semin Thromb Hemost.

[13] Prokopchuk-Gauk O, Brose K (2015). Tranexamic acid to treat life-threatening hemorrhage in prostate cancer associated disseminated intravascular coagulation with excessive fibrinolysis. Cureus.

[14] Sallah S, Gagnon GA (2000). Reversion of primary hyperfibrinogenolysis in patients with hormone-refractory prostate cancer using docetaxel. Cancer Invest.

[15] Winther Larsen A, Sandfeld-Paulsen B, Hvas AM (2020). Hyperfibrinolysis in patients with solid malignant neoplasms: a systematic review. Seminars in thrombosis and hemostasis.

[16] Rickles FR, Brenner B (2008). Tissue factor and cancer. Semin Thromb Hemost.

[17] Duran I, Tannock IF (2006). Disseminated intravascular coagulation as the presenting sign of metastatic prostate cancer. J Gen Intern Med.

